# Skeletal muscle mitochondrial correlates of critical power and *W'* in healthy active individuals

**DOI:** 10.1113/EP091835

**Published:** 2024-04-09

**Authors:** Donald L. Peden, Robert Rogers, Emma A. Mitchell, Suzanne M. Taylor, Stephen J. Bailey, Richard A. Ferguson

**Affiliations:** ^1^ School of Sport, Exercise and Health Sciences Loughborough University Loughborough UK

**Keywords:** citrate synthase activity, high‐resolution respirometry, mitochondrial content, mitochondrial function, power–duration relationship, severe‐intensity exercise

## Abstract

The asymptote (critical power; CP) and curvature constant (*W'*) of the hyperbolic power–duration relationship can predict performance within the severe‐intensity exercise domain. However, the extent to which these parameters relate to skeletal muscle mitochondrial content and respiratory function is not known. Fifteen males (peak O_2_ uptake, 52.2 ± 8.7 mL kg^−1^ min^−1^; peak work rate, 366 ± 40 W; and gas exchange threshold, 162 ± 41 W) performed three to five constant‐load tests to task failure for the determination of CP (246 ± 44 W) and *W'* (18.6 ± 4.1 kJ). Skeletal muscle biopsies were obtained from the vastus lateralis to determine citrate synthase (CS) activity, as a marker of mitochondrial content, and the ADP‐stimulated respiration (_P_) and maximal electron transfer (_E_) through mitochondrial complexes (C) I–IV. The CP was positively correlated with CS activity (absolute CP, *r* = 0.881, *P* < 0.001; relative CP, *r* = 0.751, *P* = 0.001). The *W'* was not correlated with CS activity (*P *> 0.05). Relative CP was positively correlated with mass‐corrected CI + II_E_ (*r* = 0.659, *P* = 0.038), with absolute CP being inversely correlated with CS activity‐corrected CIV_E_ (*r* = −0.701, *P* = 0.024). Relative *W'* was positively correlated with CS activity‐corrected CI + II_P_ (*r* = 0.713, *P* = 0.021) and the phosphorylation control ratio (*r* = 0.661, *P* = 0.038). There were no further correlations between CP or *W'* and mitochondrial respiratory variables. These findings support the assertion that skeletal muscle mitochondrial oxidative capacity is positively associated with CP and that this relationship is strongly determined by mitochondrial content.

## Abstract (highlights)

- **What is the central question of this study?**
  The asymptote (critical power; CP) and curvature constant (*W'*) of the hyperbolic power–duration relationship are important determinants of severe‐intensity exercise performance. We assessed the relationship between these parameters and skeletal muscle mitochondrial content and respiration.
- **What us the main finding and its importance?**
  Citrate synthase (CS) activity was positively correlated with CP. Relative CP was positively correlated with mass‐corrected CI + II_E_; absolute CP was inversely correlated with CS activity‐corrected CIV_E_. CS activity was not correlated with *W'*. CS activity‐corrected CI + II_P_ and the phosphorylation control ratio were positively correlated with relative *W'*. Mitochondrial content influences CP. Some facets of mitochondrial respiration might influence *W'*.

## 1 INTRODUCTION

The hyperbolic relationship between power output and time to exhaustion during high‐intensity exercise can be described by a power asymptote, critical power (CP), and curvature constant, *W*′ (Monod & Scherrer, 1965; Moritani et al., 1981; Poole et al., 1988). The CP demarcates the heavy and severe exercise intensity domains (Jones et al., 2008; Poole et al., 1988; Whipp, 1996) and determines exercise performance capabilities within the severe‐intensity exercise domain (Jones et al., 2010; Vanhatalo et al., 2011). Both parameters can be of importance to athletes, coaches and exercise physiologists interested in fatigue development and its underpinning mechanisms (Burnley et al., 2012; Jones et al., 2010; Poole et al., 2016; Vanhatalo et al., 2010, 2011).

Critical power is considered to reflect the greatest sustainable rate of oxidative metabolism in the absence of a progressive loss of systemic and local muscle metabolic homeostasis (Jones et al., 2010). The relationship between CP and the broad physiological parameters of aerobic function, such as oxygen delivery and oxygen uptake (${\dot{V}}_{O_{2}}$) kinetics, have been well established and are underpinned, in part, by skeletal muscle morphology and by mitochondrial content and function (Derkele et al., 2012; Goulding & Marwood, 2023; Korzeniewski & Rossiter, 2021; Murgatroyd et al., 2011; Vanhatalo et al., 2010; Whipp, 1996). The skeletal muscle morphological underpinnings of CP have been explored previously. Vanhatalo et al. (2016) previously reported a positive relationship (*r* = 0.67) between CP and the proportion of type I muscle fibres in recreationally active individuals. More recently, critical torque during intermittent knee‐extensor exercise, which is analogous to CP during cycling, was shown to be positively correlated (*r* = 0.62 for relative critical torque) with myosin heavy chain type I isoform distribution (McDougall et al., 2023). We previously reported, in endurance‐trained individuals, large positive correlations between CP and skeletal muscle capillarity, particularly around type I fibres (*r* = 0.94) and type I fibre composition (*r* = 0.79) (Mitchell et al., 2018).

Given that CP is considered to be a parameter underpinned primarily by aerobic metabolism (Goulding & Marwood, 2023; Jones et al., 2019, 2010) and that the capacity for skeletal muscle oxidative metabolism is determined by the overall capacity of the mitochondria (Hoppeler et al., 1973; Van Der Zwaard et al., 2016), there is a firm bioenergetic basis for mitochondrial content and/or respiratory function to relate positively to CP. Given that increased mitochondrial density is a characteristic of type I fibres (Picard et al., 2012; Reisman et al., 2022; Sjöström et al., 1982) and that CP and type I fibre composition are positively correlated (Mitchell et al., 2018; Vanhatalo et al., 2016), it stands to reason that CP will be positively associated with mitochondrial content. Consistent with this hypothesis, McDougall et al. (2023) recently demonstrated that critical torque was correlated (*r* = 0.48–0.70) with biomarkers of mitochondrial protein content. Whether CP relates to mitochondrial respiratory parameters is uncertain. There is evidence from animals to suggest that there is a functional specialization of mitochondria from oxidative (primarily type I) muscle fibres compared with glycolytic (primarily type II) muscle fibres (Mishra et al., 2015). Although there is uncertainty about whether there are differences in respiratory function between different muscle and fibre types (Jacobs, Díaz, Soldini, et al., 2013; Picard et al., 2012), recent work demonstrated that maximal respiration through mitochondrial complexes (C) I and II was 25% higher in human type I compared with type II fibres (Edman et al., 2024). Therefore, further research is required to assess how CP relates to mitochondrial respiratory function.

In comparison to CP, the physiological basis of *W′* is less clear. Several observations have challenged the classic interpretation that *W′* represents a fixed anaerobic energy store (Morton, 2006); for instance, *W′* appears to be sensitive to changes in oxygen delivery (Vanhatalo et al., 2010). It has also been reported that *W′* is linked to the development of the ${\dot{V}}_{O_{2}}$ slow component and the attainment of critical intramuscular pH, [PCr] and [P_i_] (Jones et al., 2008; Vanhatalo et al., 2011). Although such effects might be associated with muscle fibre composition and recruitment patterns (Krustrup et al., 2004; Pringle et al., 2003), the evidence is contradictory (Zoladz et al., 2008). Vanhatalo et al. (2016) did not report any relationship between the magnitude of *W′* and the proportion of type II fibres in recreationally active individuals, but it has been reported recently that *W′* during isolated knee‐extensor exercise was positively correlated (*r* = 0.54) with myosin heavy chain IIX composition (McDougall et al., 2023). It has also been reported that *W′* is not correlated with indices of skeletal muscle capillarity in an endurance‐trained population (Mitchell et al., 2018). This suggests that *W* *′* is mediated by alternative mechanisms, but the extent to which *W* *′* is linked to mitochondrial content and/or respiration is unclear.

The aim of the present study, therefore, was to assess the relationship between parameters of the power–duration relationship (CP and *W* *′*) and indices of mitochondrial content and respiration in healthy active individuals. Specifically, the association of CP and *W* *′* with citrate synthase (CS) activity, a valid marker of mitochondrial content (Larsen et al., 2012), and mitochondrial respiration, expressed relative to tissue mass and CS activity, was investigated. It was hypothesized that CP, but not *W* *′*, would be positively related to CS activity and maximal ADP‐stimulated respiration.

## 2 MATERIALS AND METHODS

### 2.1 Ethical approval

All experimental procedures were approved by the Loughborough University Ethics Approvals Human Participants Sub‐Committee (R19‐P230) and conformed to the *Declaration of Helsinki*, except for registration in a database. Participants were fully informed of the risks and discomforts associated with all experimental trials before providing written, informed consent.

### 2.2 Participants

Fifteen healthy males (Table 1) volunteered to participate in the study. Participants ranged from being recreationally active to competitive cyclists or triathletes. All completed health and muscle biopsy screening questionnaires prior to participation to mitigate for contraindications to maximal exercise and muscle biopsy procedures. Participants did not have a history of neuromuscular, haematological or musculoskeletal abnormalities or of allergy to administration of lignocaine hydrochloride, and they were not using pharmacological treatments during the study period.

**TABLE 1:** Participant characteristics and performance parameters.

	Mean ± SD	Range
Participant characteristics		
Age (years)	24 ± 5	19–37
Height (m)	1.78 ± 0.06	1.67–1.93
Body mass (kg)	74.2 ± 9.0	60.2–90.5
Performance parameters		
${\dot{V}}_{O_{2}\mathrm{peak}}$ (L min^−1^)	3.83 ± 0.46	2.66–4.45
${\dot{V}}_{O_{2}\mathrm{peak}}$ (mL min^−1^ kg^−1^)	52.2 ± 8.7	39.4–72.4
WR_peak_ (W)	351 ± 40	259–405
WR_peak_ (W kg^−1^)	4.8 ± 0.8	4.0–6.7
CP (W)	246 ± 44	162–313
CP (W kg^−1^)	3.4 ± 0.8	2.5–5.0
*W* *′* (kJ)	18.6 ± 4.1	11.4–24.7
*W* *′* (J kg^−1^)	0.25 ± 0.05	0.13–0.33
GET_WR_ (W)	162 ± 41	90–215
GET_WR_ (W kg^−1^)	2.2 ± 0.6	1.3–3.2
$\mathrm{GE}T_{\dot{V}O_{2}}$ (L min^−1^)	2.08 ± 0.42	1.40–2.85
$\mathrm{GE}T_{\dot{V}O_{2}}$ (mL min^−1^ kg^−1^)	28.5 ± 7.4	19.5–42.0

*Note*: All measures are *n* = 15.

Abbreviations: CP, critical power; GET, gas exchange threshold; ${\dot{V}}_{O_{2}\mathrm{peak}}$, peak oxygen uptake; *W'*, curvature constant; WR_peak_, peak work rate.

### 2.3 Experimental protocol

Participants attended the laboratory on five to seven occasions over a period of ∼30 days. Peak oxygen uptake (${\dot{V}}_{O_{2}\mathrm{peak}}$), peak work rate (WR_peak_) and gas exchange threshold (GET) were tested initially. After ≥48 h, participants undertook a series of three to five constant‐load tests to the limit of tolerance, to determine CP and *W′*, each separated by a minimum of 48 h. A minimum of 48 h after the final exercise trial, a resting muscle biopsy was obtained.

All performance tests were conducted upon an electronically braked cycle ergometer (Lode Excalibur Sport, Lode BV, Gronigen, The Netherlands). Ergometer saddle and handlebar dimensions were recorded for each participant during preliminary testing and remained standardized for the remainder of the testing period. Participants were instructed to maintain a normal diet during the testing period and to refrain from ingesting alcohol and caffeine during the 48 h before testing. All tests were conducted in constant laboratory ambient conditions (19–21°C, 40%–50% humidity).

### 2.4 Performance measures

#### 2.4.1 Peak oxygen uptake, peak work rate and gas exchange threshold

Participants performed an incremental test to exhaustion to establish ${\dot{V}}_{O_{2}\mathrm{peak}}$, WR_peak_ and GET. Participants began cycling at a freely chosen constant pedal cadence at 50 W, with power increasing at a ramp rate of 30 W min^−1^ (i.e., 1 W every 2 s) until volitional exhaustion or when cadence fell 10% below the chosen cadence for >5 s, despite strong verbal encouragement. Pulmonary gas exchange was measured continuously throughout exercise (Vyntus CPX; Carefusion, San Diego, CA, USA). The ${\dot{V}}_{O_{2}\mathrm{peak}}$ was defined as the highest ${\dot{V}}_{O_{2}}$ for a 30 s period. The WR_peak_ was defined as the highest power output achieved during the test. The GET was determined using three criteria (Beaver et al., 1986): (1) the first disproportionate increase in the rate of carbon dioxide production (${\dot{V}}_{CO_{2}}$) in proportion to ${\dot{V}}_{O_{2}}$; (2) an increase in expired ventilation (V˙E)/(V˙E)V˙O2V˙O2 with no increase in V˙E/V˙EV˙CO2V˙CO2; and (3) the first increase in end‐tidal O_2_ tension with no fall in end‐tidal CO_2_. To determine the power output that evoked GET (GET_WR_) the linear interpolation method was used. All parameters were expressed as absolute (abs) and relative to body mass (rel).

#### 2.4.2 Critical power and *W′*

Participants performed a minimum of three constant‐load tests that were continued until the limit of tolerance at between 75% and 100% of WR_peak_, the sequence of which was randomized. These were designed to elicit exhaustion within 2–15 min (Jones et al., 2019). Each test was preceded with an initial warm‐up at 50 W for 5 min. The time to exhaustion (*t*) was recorded to the nearest second and was taken as either volitional exhaustion or when the pedal cadence fell 10% below the freely chosen cadence for >5 s, despite strong verbal encouragement. No feedback regarding the power output or times achieved was provided; however, participants were permitted to view the pedal cadence throughout. To enhance the accuracy of parameter estimates, when the standard error (SE) of CP was >5% and *W′* was >10%, an additional test was performed (Poole et al., 1988).

The parameters of the power–duration relationship (CP and *W′*) were calculated using the inverse linear model [Equation (1)], the linear work–time model [Equation (2)] and the hyperbolic model [Equation (3)]. The equation associated with the lowest combined SE for each participant was selected.

(1) $$P=W^{\prime }\times \left(1/t\right)+\mathrm{CP}$$

(2) $$W=\mathrm{CP}\times t+W^{\prime }$$

(3) $$t=W^{\prime }/\left(P-\mathrm{CP}\right)$$

where *P* is power output and *W* is total work done. All parameters were expressed as absolute (abs) and relative to body mass (rel).

### 2.5 Muscle sampling and analysis

Muscle biopsies were obtained, at rest, from the lateral portion of the vastus lateralis muscle under local anaesthesia (4 mL, 1% lignocaine) using the percutaneous needle biopsy technique with suction. A small (∼10 mg) portion of the sample was immediately placed in BioPS solution (see subsection 2.5.1 below) and stored on ice for high‐resolution respirometry analysis within 8 h (*n *= 10). The remainder of the sample was immediately snap‐frozen in liquid nitrogen and stored at −80°C until analysis.

#### 2.5.1 High‐resolution respirometry

Muscle samples were placed in ice‐cold BioPS (2.77 mM CaK_2_EGTA, 7.23 mM K_2_EGTA, 5.77 mM Na_2_ATP, 6.56 mM MgCl_2_, 20 mM taurine, 50 mM 2‐(*N*‐morpholino)ethanesulfonic acid, 15 mM Na_2_‐phosphocreatine, 20 mM imidazole and 0.5 mM dithiothreitol, adjusted to pH 7.1 using titrations of KOH). Under a low‐power microscope, muscle samples were dissected of connective tissue and fat before mechanical separation of two 1–3 mg muscle fibre bundles. Subsequently, the muscle fibres were quickly transferred into an ice‐cold saponin solution [50 μg/mL BioPS, 20 μL saponin stock (5 mg saponin/mL BioPS)] and gently agitated on ice for 30 min to facilitate chemically the permeabilization of the plasma membrane. The samples were then transferred into 2 mL of MiR05 (a respiration medium containing: 110 mM sucrose, 60 mM K^+^‐lactobionate, 0.5 mM EGTA, 3 mM MgCl_2_, 20 mM taurine, 10 mM KH_2_PO_4_, 20 mM HEPES, adjusted to pH 7.1 with KOH at 37°C, and 1 g/L bovine serum albumin, essentially fatty acid free) and gently agitated on ice for 10 min before being transferred into a fresh 2 mL of MiR05 to ensure that no saponin remained in the bundles or media (Pesta & Gnaiger, 2012). The muscle fibres were then dried for 5 s on filter paper, weighed and transferred into a fresh 1 mL of ice‐cold MiR05. Mitochondrial respiration was measured in duplicate after fully immersing the fibres into MiR05 at 37°C in the chamber of a high‐resolution respirometer (O2k; Oroboros, Innsbruck, Austria). Using DatLab v.7.3 software (Oroboros), the O_2_ concentration (in nanomoles per millilitre) and flux (in picomoles per second per milligram) were recorded instantaneously. To avoid any potential O_2_ diffusion limitation, the O_2_ concentration was maintained in a range of 200–500 μM (Pesta & Gnaiger, 2012). This required monitoring the O_2_ concentration and re‐oxygenating by direct syringe injection of pure O_2_ when necessary.

A substrate–uncoupler–inhibitor–titration protocol (SUIT 8) was used to determine leak respiration, ADP‐coupled oxidative phosphorylation (_P_) and electron transfer system capacity (_E_) in mitochondrial protein complexes I—IV (CI—CIV). Steady states of O_2_ flux were marked in DatLab v.7.3 (Oroboros) after titrations in the following sequence: 5 mM pyruvate and 2 mM malate were added in the absence of adenylates to measure leak (_L_) respiration through CI (CI_L_). To ensure saturating concentrations of ADP, 5 mM was titrated, before multiple titrations of 2.5 mM ADP until respiration ceased to increase (7.5–10 mM). Thereafter, 10 mM glutamate was added to determine maximum CI_P_. Next, 10 μM cytochrome *c* was added to test the intactness of the outer mitochondrial membrane, and experimental runs were omitted from analysis if an increase of >15% was observed (*n* = 3). Subsequently, 10 mM succinate was added, and after stabilization, repeated titrations of 2.5 mM ADP were administered until respiration ceased to increase (0–2.5 mM), ensuring saturating concentrations through the succinate‐linked respiratory pathway. The maximum steady‐state respiration after this step represents CI + II_P_. A series of stepwise carbonyl cyanide 4‐(trifluoromethoxy) phenylhydrazone (FCCP) titrations (0.75–1.5 mM) were then added until no further increase in O_2_ flux was observed, for assessment of CI + II_E_. Afterwards, 0.5 μM rotenone, a CI inhibitor, was added for the determination of CII_E_. Addition of 2.5 μM antimycin A, a CIII inhibitor, allowed for the measurement of, and subsequent correction for, residual O_2_ consumption (ROX), indicative of non‐mitochondrial O_2_ consumption. Next, artificial electron donors for CIV, 2 mM ascorbate and 0.5 mM *N*,*N*,*N′*,*N′*‐tetramethyl‐*p*‐phenylenediamine (TMPD), were added to measure CIV_E_. Lastly, ≥100 mM of sodium azide was added, inhibiting all mitochondrial respiration and allowing for the calculation of autoxidation of the O2k electrode, which is artificially increased following the titration of ascorbate and TMPD, to allow for the correction of CIV_E_. Chamber cleaning procedures were followed strictly before and after all analysis, according to the recommendation of the manufacturer (Oroboros), to ensure accuracy and reliability.

#### 2.5.2 Citrate synthase activity

Approximately 15–20 mg of frozen muscle tissue was homogenized in cold lysis buffer (1:10 wet weight/volume) containing PBS, 0.2% Triton X‐100, 1 mM EDTA and protease and phosphatase inhibitor cocktail (Fisher Scientific, Loughborough, UK). Samples were blitzed using a tissue lyser (Qiagen, UK) for 4 min at 20 Hz and centrifuged at 12,000*g* for 10 min to pellet insoluble material. The supernatant was then transferred to a fresh tube, and protein concentration was determined in duplicate by Pierce 660 protein assay according to the manufacturer's instructions (Fisher Scientific, Loughborough, UK). Citrate synthase activity was analysed in triplicate in a 96‐well plate. Ten microlitres of muscle homogenate (1 mg mL^−1^) was titrated into each well, which contained 40 μL of 3 mM acetyl CoA, 25 μL of 1 mM 5,5′‐dithiobis(2‐nitrobenzoic acid) (DTNB) solution to 165 μL 100 mM Tris buffer (pH 8.3) and 10 μL of 1% Triton X‐100. Immediately before placing the plate into the spectrophotometer (Varioskan Flash, Thermo Scientific, Loughborough, UK), 15 μL of 10 mM oxaloacetic acid was added to the wells. Samples were maintained at 30°C, and after 30 s of linear agitation, absorbance at 412 nm was recorded every 15 s for 3 min. Values were corrected for the path length of the plate and expressed as moles per hour per kilogram. The coefficient of variation for CS activity was 5.7 ± 6.1%.

Respirometry values are reported relative to tissue mass and CS activity. These values were also used to calculate flux control ratios (FCRs) as follows: leak control ratio (LCR), the quotient of CI_L_ over CI + II_E_; phosphorylation control ratio (PCR), the quotient of CI + II_P_ over CI + II_E_; coupling or inverse respiratory control ratio (InvRCR), the quotient of CI_L_ over CI + II_P_; substrate control ratio (SCR), the quotient of CI_P_ over CI + II_P_ at constant OXPHOS; and reserve CIV capacity (CIVres), the quotient of CI + II_P_ over CIV_E_.

### 2.6 Statistics

All statistical analysis was performed in SPSS (IBM statistics, v.29). Data were initially checked for normality using Shapiro–Wilk tests. When normally distributed, relationships were analysed using Pearson's product–moment correlation (*r*). When not normally distributed, relationships were analysed using Spearman's rank correlation (ρ). Data are displayed as the mean ± SD (and range) unless otherwise stated. Significance was accepted at *P* ≤ 0.05.

## 3 RESULTS

The ranges of times to exhaustion for the shortest and longest trials were 177–274 s (208 ± 28 s) and 344–673 s (477 ± 101 s), respectively. Power–duration relationship parameters were established from three trials (*n *= 11), four trials (*n* = 3) and five trials (*n* = 1). The inverse linear (*n* = 8) and hyperbolic (*n = *7) models provided lowest combined SEs for CP (inverse linear, 1.1 ± 1.0%; hyperbolic, 1.2 ± 0.7%) and *W′* (inverse linear, 4.0 ± 3.0%; hyperbolic, 6.1 ± 2.9%), respectively. The linear work–time model did not yield the lowest combined SE at any time. Participant and performance characteristics and mitochondrial content/function parameters are displayed in Tables 1 and 2, respectively.

**TABLE 2:** Mitochondrial parameters.

	Mean ± SD	Range
CS activity (mol h^−1^ kg^−1^)	14.5 ± 2.3	10.9–18.7
Mass‐corrected (pmol s^−1^ mg^−1^):		
CI_L_	3.48 ± 1.55	1.93–7.09
CI_P_	56.68 ± 17.91	36.60–90.28
CI + II_P_	80.05 ± 25.14	47.91–131.71
CI + II_E_	91.61 ± 21.96	67.31–134.08
CII_E_	39.47 ± 8.22	26.87–54.19
CIV_E_	212.89 ± 41.82	150.24–289.19
CS activity‐corrected (pmol s^−1^ CS^−1^):		
CI_L_	0.25 ± 0.11	0.13–0.47
CI_P_	4.00 ± 1.11	2.34–5.87
CI + II_P_	5.64 ± 1.50	3.29–8.56
CI + II_E_	6.45 ± 1.16	4.78–8.76
CII_E_	2.80 ± 0.55	1.97–3.53
CIV_E_	15.07 ± 3.14	10.31–18.86
Flux control ratios		
LCR	0.039 ± 0.018	0.020–0.085
PCR	0.867 ± 0.118	0.573–0.982
InvRCR	0.049 ± 0.036	0.022–0.148
SCR	0.735 ± 0.258	0.549–1.444
CIVres	0.383 ± 0.119	0.220–0.580

*Note*: All measures are *n* = 10, except CS activity, which is *n* = 15.

Abbreviations: CI_L_, leak respiration through mitochondrial protein complex (C) I of the electron transfer system; CI_P_ and CI + II_P_, ADP‐stimulated respiration (_P_) through CI and CII of the electron transfer system, respectively; CI + II_E_, CII_E_ and CIV_E_, maximal non‐coupled electron transfer system capacity (_E_) of CI and CII, CII and CIV, respectively; CIVres, reserve capacity of mitochondrial protein complex IV, the quotient of CI + II_P_ over CIV_E_; CS, citrate synthase; InvRCR, inverse respiratory control ratio the quotient of CI_L_ over CI + II_P_; LCR, leak control ratio, the quotient of CI_L_ over CI + II_E_; PCR, phosphorylation control ratio, the quotient CI + II_P_ over CI + II_E_; SCR, substrate control ratio, the quotient of CI_P_ over CI + II_P_ at constant oxidative phosphorylation.

### 3.1 Correlates of CP

**FIGURE 1:**
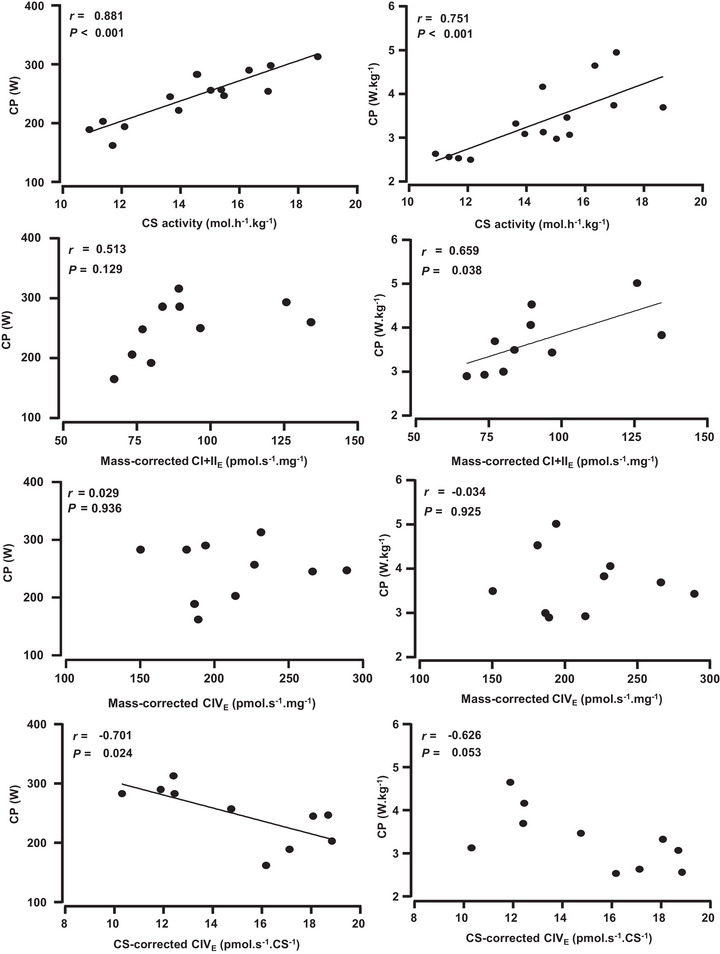
Correlations between CP (absolute and relative) and CS activity, mass‐corrected CI + II_E_ and CIV_E_ and CS activity‐corrected CIV_E_. Abbreviations: CI + II_E_ and CIV_E_, maximal non‐coupled electron transfer system capacity (_E_) of mitochondrial complexes (C) I and CII, and CIV, respectively; CP, critical power; CS, citrate synthase.

Correlations between CP and CS activity and mitochondrial respiration parameters are shown in Table 3 and Figure 1. Absolute and relative CP were positively correlated with CS activity. Relative CP was correlated with mass‐corrected CI + II_E_. Absolute and relative CP were not correlated with any other mass‐corrected mitochondrial respiratory parameters. Absolute CP was inversely correlated with CS activity‐corrected CIV_E_. Absolute and relative CP were not correlated with any other CS activity‐corrected mitochondrial respiratory parameters There were no correlations between CP and any FCR parameters.

**TABLE 3:** Correlations between critical power (absolute and relative) and citrate synthase activity, mass‐corrected mitochondrial function, citrate synthase activity‐corrected mitochondrial function and flux control ratios.

	CP (W)	CP (W kg^−1^)
CS activity (mol h^−1^ kg^−1^)	*r* = 0.881, *P* < 0.001	*r* = 0.751, *P* = 0.001
Mass‐corrected (pmol s^−1^ mg^−1^):		
CI_L_	*r *= 0.395, *P* = 0.259	*r *= 0.161, *P* = 0.657
CI_P_	*r *= 0.373, *P* = 0.288	*r *= 0.441, *P* = 0.202
CI + II_P_	*r *= 0.295, *P* = 0.407	*r *= 0.545, *P* = 0.103
CI + II_E_	*r *= 0.513, *P* = 0.129	*r *= 0.659, *P* = 0.038
CII_E_	*r *= 0.354, *P* = 0.315	*r *= 0.116, *P* = 0.750
CIV_E_	*r *= 0.029, *P* = 0.936	*r *= −0.034, *P* = 0.925
CS activity‐corrected (pmol s^−1^ CS^−1^):		
CI_L_	*r *= 0.088, *P* = 0.810	*r *= −0.099, *P* = 0.785
CI_P_	*r *= −0.076, *P* = 0.834	*r *= 0.055, *P* = 0.880
CI + II_P_	*r *= −0.213, *P* = 0.555	*r *= 0.148, *P* = 0.683
CI + II_E_	*r *= −0.120, *P* = 0.741	*r *= 0.163, *P* = 0.633
CII_E_	*r *= −0.383, *P* = 0.275	*r *= −0.506, *P* = 0.136
CIV_E_	*r *= −0.701, *P* = 0.024	*r *= −0.626, *P* = 0.053
Flux control ratios		
LCR	ρ = −0.067, *P* = 0.854	ρ = −0.187, *P* = 0.606
PCR	ρ = −0.389, *P* = 0.266	ρ = −0.195, *P* = 0.589
InvRCR	ρ = −0.006, *P* = 0.987	ρ = −0.086, *P* = 0.814
SCR	ρ = −0.036, *P* = 0.920	ρ = −0.061, *P* = 0.867
CIVres	*r *= 0.291, *P* = 0.415	*r *= 0.605, *P* = 0.064

*Note*: All measures are *n* = 10, except CS activity, which is *n* = 15.

Abbreviations: CI_L_, leak respiration through mitochondrial protein complex (C) I of the electron transfer system; CI_P_ and CI + II_P_, ADP‐stimulated respiration (_P_) through CI and CII of the electron transfer system, respectively; CI + II_E_, CII_E_ and CIV_E_, maximal non‐coupled electron transfer system capacity (_E_) of CI and CII, CII and CIV, respectively; CIVres, reserve capacity of mitochondrial protein complex IV, the quotient of CI + II_P_ over CIV_E_; CP, critical power; CS, citrate synthase; InvRCR, inverse respiratory control ratio the quotient of CI_L_ over CI + II_P_; LCR, leak control ratio, the quotient of CI_L_ over CI + II_E_; PCR, phosphorylation control ratio, the quotient CI + II_P_ over CI + II_E_; SCR, substrate control ratio, the quotient of CI_P_ over CI + II_P_ at constant oxidative phosphorylation.

### 3.2 Correlates of *W* *′*

**FIGURE 2:**
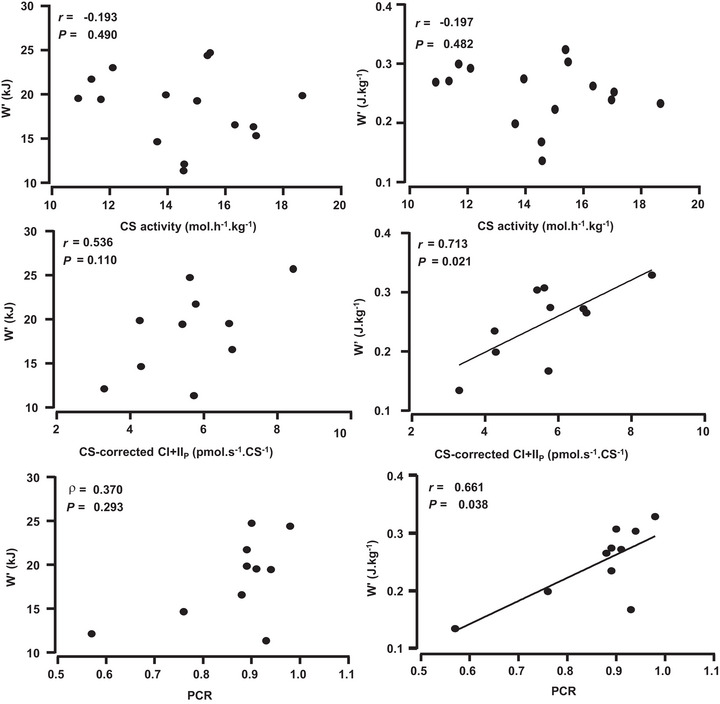
Correlations between *W'* (absolute and relative) and CS activity, CS activity‐corrected CI + II_P_ and PCR. Abbreviations: CI + II_P_, ADP‐stimulated respiration (_P_) through mitochondrial complexes (C) I and CII of the electron transfer system; CS, citrate synthase; PCR, phosphorylation control ratio, the quotient CI + II_P_ over CI + II_E_; *W'*, curvature constant.

Correlations between *W′* and CS activity and mitochondrial respiration parameters are shown in Table 4 and Figure 2. There were no correlations between absolute or relative *W′* and CS activity or any parameters of mass‐corrected mitochondrial function. There was a positive correlation between relative *W′* and CS activity‐corrected CI + II_P_, although this was not evident for absolute *W′*. There was a positive correlation between relative *W′* and PCR, although this was not evident for absolute *W′*. Absolute (*r* = −0.663; *P *= 0.037) and relative (*r *= −0.748; *P *= 0.013) *W′* were inversely correlated with InvRCR, although these were skewed by a severe outlier, which, when omitted, removed the significant correlations (Table 4).

**TABLE 4:** Correlations between *W'* (absolute and relative) and citrate synthase activity, mass‐corrected mitochondrial function, citrate synthase activity‐corrected mitochondrial function and flux control ratios.

	*W'* (kJ)	*W'* (kJ kg^−1^)
CS activity (mol h^−1^ kg^−1^)	*r* = −0.193, *P* = 0.490	*r* = −0.197, *P* = 0.482
Mass‐corrected (pmol s^−1^ mg^−1^):		
CI_L_	*r *= −0.472, *P* = 0.168	*r *= −0.561, *P* = 0.092
CI_P_	*r *= 0.113, *P* = 0.757	*r *= 0.167, *P* = 0.645
CI + II_P_	*r *= 0.454, *P* = 0.187	*r *= 0.558, *P* = 0.094
CI + II_E_	*r *= 0.259, *P* = 0.469	*r *= 0.318, *P* = 0.371
CII_E_	*r *= 0.448, *P* = 0.194	*r *= 0.263, *P* = 0.463
CIV_E_	*r *= 0.564, *P* = 0.090	*r *= 0.425, *P* = 0.220
CS activity‐corrected (pmol s^−1^ CS^−1^):		
CI_L_	*r *= −0.454, *P* = 0.188	*r *= −0.507, *P* = 0.135
CI_P_	*r *= 0.134, *P* = 0.711	*r *= 0.243, *P* = 0.499
CI + II_P_	*r *= 0.536, *P* = 0.110	*r *= 0.713, *P* = 0.021
CI + II_E_	*r *= 0.358, *P* = 0.310	*r *= 0.513, *P* = 0.130
CII_E_	*r *= 0.495, *P* = 0.146	*r *= 0.393, *P* = 0.261
CIV_E_	*r *= 0.574, *P* = 0.083	*r* = 0.545, *P* = 0.104
Flux control ratios		
LCR	ρ = −0.426, *P* = 0.220	ρ = −0.389, *P* = 0.266
PCR	ρ = 0.370, *P* = 0.293	ρ = 0.661, *P* = 0.038
InvRCR	ρ = −0.552, *P* = 0.123	*r *= −0.653, *P* = 0.057
SCR	ρ = −0.152, *P* = 0.676	ρ = −0.042, *P* = 0.907
CIVres	*r *= 0.087, *P* = 0.812	*r *= 0.283, *P* = 0.428

*Note*: All measures are *n* = 10, except CS activity, which is *n* = 15, and InvRCR, which is *n* = 9.

Abbreviations: CI_L_, leak respiration through mitochondrial protein complex (C) I of the electron transfer system; CI_P_ and CI + II_P_, ADP‐stimulated respiration (_P_) through CI and CII of the electron transfer system, respectively; CI + II_E_, CII_E_ and CIV_E_, maximal non‐coupled electron transfer system capacity (_E_) of CI and CII, CII and CIV, respectively; CIVres, reserve capacity of mitochondrial protein complex IV, the quotient of CI + II_P_ over CIV_E_; CS, citrate synthase; InvRCR, inverse respiratory control ratio the quotient of CI_L_ over CI + II_P_; LCR, leak control ratio, the quotient of CI_L_ over CI + II_E_; PCR, phosphorylation control ratio, the quotient CI + II_P_ over CI + II_E_; SCR, substrate control ratio, the quotient of CI_P_ over CI + II_P_ at constant oxidative phosphorylation; *W'*, curvature constant.

## 4 DISCUSSION

The main findings of this study are as follows: (1) CS activity (a valid marker of mitochondrial content) was positively correlated with both absolute and relative CP. Furthermore, although mass‐corrected CI + II_E_ was positively correlated with relative CP, this relationship was no longer evident when controlling for CS activity; (2) there was no correlation between CS activity and *W′*; and (3) CS activity‐corrected CI + II_P_ and PCR were positively correlated with relative *W′*.

### 4.1 Correlates of CP

The primary finding of this study was the positive correlation between CS activity and both absolute and relative CP. Citrate synthase activity is accepted to be a valid biomarker of mitochondrial content (Larsen et al., 2012), with a large correlation coefficient *(r =* 0.84) and a substantial concordance (*Rc *= 0.80) with the gold‐standard technique of transmission electron microscopy. These new findings extend previous observations of large positive correlations between CP and indices of skeletal muscle fibre type (Mitchell et al., 2018; Vanhatalo et al., 2016) and capillarity (Mitchell et al., 2018).

The positive association between CP and mitochondrial content is a new finding. Critical power is higher in endurance‐trained populations (Jones et al., 2010), and greater mitochondrial content is observed in endurance‐trained individuals (Jacobs & Lundby, 2013; Moore et al., 1987; Rimbert et al., 2004; Roepstorff et al., 2005; Russell et al., 2002; Zoll et al., 2002). Increased mitochondrial density is a characteristic of type I fibres (Picard et al., 2012; Reisman et al., 2022; Sjöström et al., 1982). Additionally, capillary density is also highly correlated with mitochondrial density and the proportion of oxidative fibres (Poole et al., 1991; Schwerzmann et al., 1989). These associations support the relationship between mitochondrial density and CP observed in the present study. The high correlations between CP and CS activity in the present study (*r* = 0.881) extend the moderate correlations (*r* = 0.48–0.70) found between protein markers of mitochondrial content and critical torque in isolated knee‐extensor exercise (McDougall et al., 2023). As such, the strong relationship between CP and CS activity in the present study corroborates that mitochondrial content is a key correlate of the maximal metabolic steady state, perhaps even more so in whole‐body exercise than in an isolated limb. Another explanation for this divergence might be attributable to the biomarkers used for the determination of mitochondrial content, with CS activity demonstrated to correlate better with the gold standard technique (Larsen et al., 2012).

Relative CP was positively correlated with mass‐corrected CI + II_E_, which represents the maximal electron flow through the respiratory system. This can serve as an indicator of the maximal attainable membrane potential and is the best respiratory correlate of maximal oxygen consumption (Jacobs & Lundby, 2013). However, this relationship was no longer evident when normalizing CI + II_E_ to CS activity. This also indicates that the relationship between maximal capacity of the electron transfer system and CP is determined by both the content and the function of the mitochondria. There are mixed reports regarding the effects of fibre type on ADP‐stimulated respiration and maximal uncoupled respiration in permeabilized skeletal muscle (Jacobs, Díaz, Meinild, et al., 2013; Picard et al., 2012). If the relationship between CP and mitochondrial content in the present study is fibre‐type dependent as proposed, this might not then be expected to be congruent with alterations in mitochondrial respiration. However, this speculation requires further investigation.

Despite there being no relationship when corrected to mass, CS‐corrected CIV_E_ became inversely correlated with CP. This finding might seem counter‐intuitive, given that CIV enzyme activity and protein content have been reported to be strongly associated with muscle oxidative capacity and of mitochondrial content (Larsen et al., 2012). The hypothesis that CP is inherently associated with mitochondrial content could explain this finding. When expressing CIV_E_ per mitochondrion (CS‐activity corrected) as opposed to mass, the relationship between CS activity and CP previously described is essentially inverted.

Taken together, these data suggest that CP is influenced by mitochondrial content, with some modest influence of mitochondrial respiration, in keeping with the concept that a critical exercise intensity is linked to that at which an oxidative steady state is attainable (Poole et al., 2016). A high mitochondrial content will increase the capacity for oxygen extraction (Hoppeler et al., 1973), which, combined with greater oxygen delivery afforded by greater capillarity (Hudlicka & Brown, 2009; Mitchell et al., 2018), will increase the overall capacity to utilize oxygen and buffer anaerobic byproducts (Robergs et al., 2004), contributing to greater performance capability. Increased mitochondrial density is a characteristic of type I fibres (Picard et al., 2012; Reisman et al., 2022; Sjöström et al., 1982). Accordingly, the data of the present study indicate that mitochondrial content might be the mediating variable of the relationship between CP and type I fibre composition (Mitchell et al., 2018; Vanhatalo et al., 2016).

The lack of correlation between CP and some of the mitochondrial respiratory variables is perhaps surprising. A consideration for this finding might be the uncertainty regarding whether there are differences in respiratory function, in contrast to the differences in mitochondrial content, between different fibre types (Jacobs, Díaz, Soldini, et al., 2013; Picard et al., 2012). Some evidence from isolated mitochondria and non‐invasive in vivo measures in animal models indicates differences in mitochondrial function and respiration across muscle types (Amara et al., 2007; Conley et al., 2007; Jackman & Willis, 1996; Mishra et al., 2015; Picard et al., 2008), with others indicating no differences in fibre type (Leary et al., 2003; Schwerzmann et al., 1989; Yajid et al., 1998). Observations in mouse and human permeabilized muscle fibres are likewise equivocal. Research has shown greater phosphorylation in soleus muscle (with a high proportion of type I fibres) compared with gastrocnemius and quadriceps muscle (with a low proportion of type I fibres) when normalized to mass, but not mitochondrial content (Jacobs, Díaz, Meinild, et al., 2013). Recent work demonstrated that maximal complex I and II respiration was 25% higher in human type I compared with type II fibres (Edman et al., 2024). This is in contrast to research finding no differences in phosphorylation across fibre type (Bahi et al., 2005; Ponsot, 2005). These studies highlight the importance of methodology and normalization in the interpretation of mitochondrial respirometry (Jacobs, Díaz, Soldini, et al., 2013). In addition to these methodological considerations, the intricate relationship between mitochondrial content and respiration in determining overall oxidative capacity could contribute to the variability of these findings and the lack of relationship between mitochondrial respiration and CP of the present study.

### 4.2 Correlates of *W′*

There was no correlation between *W′* and CS activity, suggesting that mitochondrial content is not a primary determinant of this parameter of the power–duration relationship. This is perhaps not surprising given that *W′* has been defined as the finite capacity for work above CP, or a fixed anaerobic energy store (Morton, 2006), although it has been characterized more recently as a fatigability constant (i.e., the buffer available to resist fatigue above CP) (Goulding et al., 2021; Poole et al., 2016). Interestingly, when controlling for mitochondrial content, CS‐corrected CI + II_P_ was positively correlated with relative *W'*. Furthermore, PCR was also positively correlated with relative *W'*. The PCR represents the maximal capacity for mitochondrial oxidative phosphorylation relative to CI + II_E_. Improvements in maximal oxidative phosphorylation have been demonstrated to be positively correlated with improvements in time‐trial performance during severe‐intensity exercise and maximal oxygen uptake (Daussin et al., 2008). Exercise in this intensity domain requires significant contributions from anaerobic energy metabolism and recruitment of less‐efficient type II muscle fibres (Poole & Jones, 2012). Therefore, it is reasonable to speculate that if aspects of oxidative phosphorylation are greater, this would result in sparing of the anaerobic energy contribution during intense exercise and impact the overall work capacity. The maximal oxidative phosphorylation and buffering capacity of the mitochondria are inherently linked to the effective utilization of anaerobic capacity (Robergs et al., 2004), which supports the notion that *W'* is not purely anaerobic in nature. However, a moderate positive correlation between myosin heavy chain IIX composition and *W'* during intermittent knee‐extensor exercise (McDougall et al., 2023) provides an alternative perspective. Therefore, the physiological underpinning of *W'* remains uncertain and in need of further investigation.

### 4.3 Limitations

This study is not without limitations. To verify the correlations between mitochondrial content and CP, additional measures of mitochondrial content could have been used. Citrate synthase activity is a valid marker, but not the gold‐standard method for the determination of mitochondrial content (Larsen et al., 2012). Furthermore, cristae density can also explain different respiratory capabilities in skeletal muscle (Schytz et al., 2004), and more comprehensive assessment of mitochondrial characteristics would allow for greater insight into the specific physiological mechanism underpinning CP. Additionally, the validity of methods examining *ex vivo* markers of mitochondrial respiration with in vivo measures of metabolic steady state must be considered. Recent research has suggested that correction for temperature of working muscle when using high‐resolution respirometry might better represent the mitochondrial capacity at a given exercise intensity, where cellular temperature is likely to exceed that of the respirometer (Jacobs & Lundby, 2021). The range of test duration for the CP prediction trials was narrower than recommended in several participants, which might have resulted in erroneous parameter estimates. Lastly, it must be considered that the sample size might have resulted in data being underpowered for some of the respirometry variables examined, given the variability of this technique.

## 5 CONCLUSIONS

This study has demonstrated a large positive correlation between CP and CS activity (a valid marker of mitochondrial content). Although mass‐corrected CI + II_E_ was positively correlated with relative CP, this relationship was no longer evident when controlling for CS activity, and there were no further correlations with mitochondrial respiratory variables. Therefore, the present study suggests that CP was more likely to be determined by mitochondrial content, rather than intrinsic mitochondrial respiration. Citrate synthase activity was not correlated with *W'*; however, CS activity‐corrected CI + II_P_ and PCR were positively correlated with relative *W'*, suggesting that some facets of mitochondrial respiration might influence *W'*.

## AUTHOR CONTRIBUTIONS

Donald L. Peden, Robert Rogers, Stephen J. Bailey and Richard A. Ferguson contributed to the conception and design of the experiment. All authors contributed to the acquisition, analysis and interpretation of data and drafting or critical revision of the manuscript. All authors approved the final manuscript and agree to be accountable for all aspects of the work in ensuring that questions related to the accuracy or integrity of any part of the work are appropriately investigated and resolved. All persons designated as authors qualify for authorship, and all those who qualify for authorship are listed.

## CONFLICT OF INTEREST

The authors declare no conflicts of interest.

## Data Availability

The raw data supporting the conclusions of this research are available from the corresponding author upon reasonable request.
